# Barriers and facilitators in accessing dementia care by ethnic minority groups: a meta-synthesis of qualitative studies

**DOI:** 10.1186/s12888-017-1474-0

**Published:** 2017-08-30

**Authors:** Cassandra Kenning, Gavin Daker-White, Amy Blakemore, Maria Panagioti, Waquas Waheed

**Affiliations:** Division of Population Health, Health Services Research and Primary Care, School of Health Sciences, The University of Manchester, 5th floor, Williamson Building, Oxford Road, Manchester, M13 9PL UK

**Keywords:** Dementia, Barriers, Facilitators, Access, Review, Meta-ethnography, Ethnicity, Minority

## Abstract

**Background:**

It is estimated that there are about 25,000 people from UK ethnic minority groups with dementia. It is clear that there is an increasing need to improve access to dementia services for all ethnic groups to ensure that everyone has access to the same potential health benefits. The aim was to systematically review qualitative studies and to perform a meta-synthesis around barriers and facilitators to accessing care for dementia in ethnic minorities.

**Methods:**

Databases were searched to capture studies on barriers and facilitators to accessing care for dementia in ethnic minorities. Analysis followed the guidelines for meta-ethnography. All interpretations of data as presented by the authors of the included papers were extracted and grouped into new themes.

**Results:**

Six hundred and eighty four papers were identified and screened. Twenty eight studies were included in the meta-synthesis. The analysis developed a number of themes and these were incorporated into two overarching themes: ‘inadequacies’ and ‘cultural habitus’.

**Conclusions:**

The two overarching themes lend themselves to interventions at a service level and a community level which need to happen in synergy.

**Trial registration:**

The review was registered with PROSPERO: CRD42016049326.

**Electronic supplementary material:**

The online version of this article (10.1186/s12888-017-1474-0) contains supplementary material, which is available to authorized users.

## Background

A study looking at global prevalence of dementia has suggested that age-specific prevalence of dementia varies little between world regions [1]. Ethnic minorities comprise approximately 34% of the population in the US [2], and 14% of the total population in the UK [3]. Currently, it is estimated that there are about 25,000 people from UK ethnic minority groups with dementia but these figures are thought to be underestimated [3]. In the UK, this means one person in every 88 (1.1%) of the entire UK population having some form of dementia, costing the UK economy £23 billion per year. As a result the cost of dementia to the economy is greater than that for heart disease and cancer combined [4]. Incidence has been forecast to increase by 38% over the next 15 years and 154% over the next 45 years [5].

However, there may also be some evidence that rates of dementia are decreasing in some high-income countries. A recent study comparing prevalence of dementia in the US between 2000 and 2012 suggests that prevalence of dementia has actually declined significantly in this period [6]. Similarly in the UK a 20% drop in the overall dementia incidence rate from 1991 to 2015 was reported [7]. Dementia remains a global problem which represents major emotional and financial burden for patients, relatives and health authorities.

It is clear that there is an increasing need to improve access to dementia services for all ethnic groups to ensure that everyone has access to the same potential health benefits [8]. Many characteristics are common to ethnic populations, for example, South Asians represent a large diverse group where accurate assessment of dementia and access to appropriate care is challenging due to low English literacy, small number of therapists with clinical skills, lack of recognition and costs to cover provision of extra resources like transport and interpreters [8, 9].

There is a growing body of evidence for the need to intervene to improve help-seeking and promote early diagnosis of dementia in minority ethnic groups [8–10]. Many countries are now carrying out educational campaigns around dementia [8]. However, there are as yet no published RCTs that show if outreach with these groups improves help-seeking and early diagnosis. Past research and reviews have only focused on bringing together barriers and facilitators as themes. However, there is a need to understand the relationships between these themes with an aim to align them with future intervention approaches and implementation studies.

As a step in this direction we propose meta-synthesis to assist knowledge synthesis through a process of re-conceptualisation of themes across included qualitative studies [11]. During the meta synthesis, the interpretations and explanations in the included studies undergo a process of deconstruction, translation and reconstruction as `a means to grasp the particulars within the wholes’ [12]. The evolving explanatory framework can then be applied to the specific aims of this review to understand barriers and facilitators to accessing dementia care by ethnic minority groups. To our knowledge this has not been done previously.

This systematic review and meta-synthesis aims to systematically explore views and experiences of ethnic minority patients, carers and health professionals on barriers and facilitators in accessing dementia care, with the purpose of discussing how such findings could contribute to the design of efficient interventions to improve access to dementia care for ethnic minority groups.

## Methods

### Literature search

The following electronic databases were searched: Cochrane Central Register of Controlled Trials (CENTRAL) 31March 2016, MEDLINE (Ovid) from inception to 3 April 2016, PsycINFO (Ovid) from inception to 3 April 2016, Embase (Ovid) from inception to 4 April2016, Cochrane Database of Systematic Reviews 2005 to 4 April 2016. In addition we reviewed reference lists of included studies and systematic reviews to identify any relevant papers not identified in the original search.

Searches were conducted to specifically capture studies on barriers and facilitators in accessing care for dementia in ethnic minorities. Search terms included: dementia, Alzheimer’s Disease; ethnic*, Asian, Black, African, minority. Searches using these terms identified around 6500 papers. To further limit the search we introduced criteria specifying qualitative research. These included: qualitative, interview, focus group*, content analysis, discourse, grounded theory, ethnograph*.

The search is available in Additional file 1.

### Eligibility criteria

Studies were eligible for inclusion in this review if they met the following criteria:

- Population: Ethnic minority patients with dementia or memory problems; carers of ethnic minority patients with dementia; health professionals working with ethnic minority patients.
- Outcomes: The qualitative analysis described barriers and enablers to ethnic minority patients accessing dementia care, or carers and health professionals’ views of barriers in accessing dementia care.
  Setting/context: Any country without restriction in publication date and language.
- Study design: Empirical studies published as peer-reviewed journal articles or conference papers which report qualitative methods of data collection and analysis. An operational definition of this criterion was that studies used semi-structured interview data and content analysis reports were included in this classification. Mixed method studies were eligible if the qualitative data were analysed separately to the quantitative data. Unpublished dissertations and book chapters were excluded.

### Study selection and data extraction

Titles and abstracts were screened by author (CK) and full papers of potentially relevant abstracts were obtained. A second researcher cross-checked and agreed the included and excluded papers (MP), final decisions about inclusion were made by the CI (WW) if there was no consensus.

Data were extracted by the author (CK) onto a standardised Excel spreadsheet. Data was collected on study details including context and participants, design and methods, as well as author’s interpretations of their data. A 50% sample of the papers were double extracted by two independent reviewers to ensure reliability of the data extraction.

### Critical appraisal of study quality

Two independent reviewers appraised the identified studies using a checklist adapted from the Critical Appraisal Skills Programme (CASP) qualitative checklist [13]. We did not exclude papers based on quality.

### Translation and synthesis

Analysis followed the guidelines for meta-ethnography outlined by Noblit and Hare [11]. Three order constructs were generated. First order constructs are defined as direct participant quotes reported in the papers. Second order constructs are defined as the authors’ interpretations of participants’ quotes expressed as themes, extracted from both the results and discussion sections of papers in order to capture all constructs. Third order constructs refer to synthesised constructs that emerge from the analysis of first and second order constructs [13]. Papers were read and re-read by two reviewers and first and second order constructs were extracted and managed using Microsoft Excel. Reviewers (CK, GDW, AB) independently sifted the second order constructs, and group discussions were held to compile new third order constructs that summarised and encompassed the various themes across study findings.

For analysis, all interpretations of data presented by the authors of the included papers were extracted. The aim was to see how previous authors had interpreted and presented the results of their studies. Authors comments that interpreted their data were extracted in to an excel spread sheet. The extracted data were then grouped into themes independently by two further reviewers (GDW, AB). Their groupings were then compared and discussed by the team to ensure consensus. Once the themes had been broadly agreed the first author read through the data in each of the themes checking that the interpretation of the data was correct and suggesting changes based on the original context of the studies. The data was read and re-read to develop new concepts which incorporated the different interpretations of the themes.

The results are reported as per PRISMA guidelines for systematic reviews.

## Results

### Search results

**Fig. 1:**
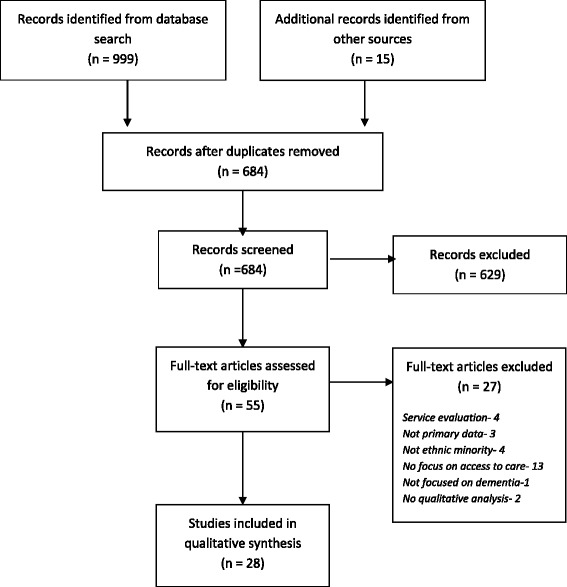
PRISMA diagram

Figure 1 shows the processes of selection. The initial search identified 999 records with a further 15 identified from reference list searches. After duplicates were removed, 684 records remained and the titles and abstracts were read to identify relevant papers. Six hundred and twenty nine records were excluded at this stage and 55 papers were selected and fully screened to establish if the inclusion criteria were met. Two further papers were excluded at the data extraction phase due to lack of useable data. In total 28 papers were included in the review (Table 1).

**Table 1:** Included papers

Study ID no.	Study	Country (urban/ rural)	Type (no.) participants	Experience/type of dementia	Ethnic group(s)	Data collection method/ dates	Method of analysis
1	Fox et al., 1999 [21]	USA (Urban)	Caregivers (10)	Caregivers of elders with diagnosis of dementia. No indication of type or severity	African American sub-sample from Levkoff et al. 1999 (below)	Interviews Dates not reported	Narrative review
2	Hicks & Lam, 1999 [22]	USA (Urban)	Caregivers (7)	Caregivers of elders with diagnosis of dementia. No indication of type or severity	Chinese American sub-sample from Levkoff et al. 1999 (below)	Interviews Dates not reported	Narrative review
3	Levkoff et al., 1999 [23]	USA (Urban)	Caregivers (40)	Caregivers of elders with diagnosis of dementia. No indication of type or severity	African American, Chinese American, Puerto Rican,Irish American	Interviews Dates not reported	Thematic analysis
4	Adamson, 2001 [24]	UK (Urban)	Caregivers (30)	Caregivers of persons with symptoms of dementia. No indication of type or severity	African/Caribbean, South Asian (Pakistan, India, East Africa)	Interviews Dates not reported	Constant comparative techniques of grounded theory
5	Karner & Hall, 2002 [25]	USA (Urban)	Service providers (42)	na	na	Interviews 1999	Grounded theory
6	Bowes & Wilkinson, 2003 [26]	UK (Urban)	Patients /Caregivers (4)Service providers (11)	Patients with diagnosis of dementia and their families/caregivers. No indication of type or severity	South Asian	Interviews& case studies Dates not reported	Thematic analysis
7	Cloutterbuck & Mahoney, 2003 [27]	USA (Urban)	Caregivers (7)	Family caregivers of elders with dementia. No indication of type or severity.	African American	Focus group Dates not reported	Content analysis
9	St. John, 2004 [28]	UK (not specified)	Community members, carers and older people (no. not reported) Service providers and managers (no. not reported)	No details on experience of dementia. No indication of type or severity	nr	Interviews & focus groups 2002–4	Content analysis categorised using a grounded theory approach
10	Beattie et al., 2005 [29]	UK (rural and urban)	Service managers & care staff (61)	na	na	Interviews 2000–2	Comparative textual analysis
11	Neary & Mahoney, 2005 [30]	USA (not specified)	Caregivers (11)	Current or recent experience of caring for a relative with dementia. 6 people were being cared for at home, 3 had moved to nursing homes. No indication of type or severity.	Colombian, Puerto Rican, Argentinian, Guatemalan,Cuban, Dominican heritage.	Interviews Dates not reported	Ethnonursing data analysis
13	Jett, 2006 [31]	USA (Urban)	General population (14)	Participants ‘knew someone with dementia’ No indication of type or severity.	African American	Interviews Dates not reported	Ethnographic approach using Grounded theory
14	Jones et al., 2006 [32]	USA (Urban)	General population (62)	People with a range of knowledge and experience regarding Alzheimer’s Disease	Japanese American, Korean American, Chinese American	Focus groups 2001–2	content analysis
15	Mackenzie, 2006 [33]	UK (Urban)	Caregivers (20)	Caregivers of relatives with dementia. No indication of type or severity.	Pakistani, Indian, Polish, Ukrainian	Interviews 2001–2	Content analysis
16	La Fontaine et al., 2007 [34]	UK (not specified)	General public (49)	No details provided on experience or knowledge of dementia.	Indian	Focus groups 2001–3	Thematic analysis
17	Vickrey et al., 2007 [35]	USA (Urban)	Caregivers (47)	Informal caregivers of people with dementia. Data on severity and duration of diagnosis for 44 participants 8 = mild, 18 = moderate, 18 = severe	African American, Chinese American, Euro American, Hispanic American	Focus groups 1998–9	Content analysis
18	Lawrence et al., 2008 [36]	UK (Urban)	Caregivers (32)	Carers of people with dementia. No indication of type or severity.	Black Caribbean, South Asian, White British	Interviews Dates not reported	Grounded theory
19	Morhardt et al., 2010 [37]	USA (Urban)	Caregivers (48)	Carers of people with “Alzheimer’s Disease and related dementias” No indication of type or severity.	Assyrian, Arab, Bosnian, Hindi,Urdu (language speakers)	Interviews & observations 2005–8	Grounded theory
20	Boughtwood et al., 2011 [38]	Australia (not specified)	Multicultural community link workers (24)	Extensive experience of and in-depth knowledge of family care-giving for dementia.	na	Interviews Dates not reported	Thematic analysis
21	Lawrence et al., 2011 [39]	UK (Urban)	Older people with dementia (30)	Dementia severity: MMSE < 11 *n* = 8 (severe) MMSE 11–20 *n* = 9 (moderate) MMSE >20 *n* = 13 (mild)	Black Caribbean, South Asian, White British older people	Interviews Dates not reported	Grounded theory
22	Mukadam et al., 2011 [40]	UK (Urban)	Caregivers (18)	“caring for people at different stages of dementia” no further details reported	White British, South Asian, Black (African or Caribbean), White Irish, White Other, Asian Other, Chinese	Interviews Dates not reported	Thematic analysis
23	Koehn et al., 2012 [41]	Canada (not specified)	Caregivers (11) and people with dementia (10) dyads	Patients with “probable ADRD”. Diagnoses made 1–4 years prior to interview	Chinese-Canadian	Interviews 2008	Critical-constructionist approach
24	Shanley et al., 2012 [42]	Australia (Urban)	Caregivers (121)Multicultural health workers (60)	Family carers with recent experience of caring for a family member with dementia. No indication of type or severity	Arabic, Chinese, Italian, Spanish (language speakers)	Focus groups (carers) & interviews (health workers) Dates not reported	Thematic analysis
25	Boughtwood et al., 2013 [43]	Australia (not specified)	Multicultural workers (24)	Employed to promote access to dementia services in CALD communities.	na	Interviews Dates not reported	Content and thematic analyses
26	Haralambous et al., 2014 [44]	Australia (not specified)	Caregivers (13)Community workers (11)Health professionals (39)	Carers of people with dementia. No indication of type or severity	Chinese, Vietnamese	Interviews & focus groups Dates not reported	Thematic analysis.
27	Uppal et al., 2014 [45]	UK (not specified)	General public (28)	No information provided if caregivers or not. No indication of type or severity	Sikh	Focus groups Dates not reported	Constant comparative methodology
28	Casado et al., 2015 [46]	USA (Urban)	Caregivers 23	Care-givers of people with dementia symptoms. No indication of type or severity	Korean American	Focus groups Dates not reported	Thematic analysis
29	Jutlla, 2015 [47]	UK (Urban)	Carers (12)	Carers of a family member with dementia. No indication of type or severity	Sikh	Interviews Dates not reported	Constructivist grounded theory
30	Mukadam et al., 2015 [48]	UK (Urban)	General public (53)	People without a known diagnosis of dementia, with or without experience of caring for people with dementia	South Asian	Focus groups & interviews Dates not reported	Interpretative phenomenological analysis

#### Quality assessments

An *a priori* decision was made that every paper meeting the inclusion criteria would be included in the review. While quality is an important consideration for experimental studies, application of quality criteria to qualitative research has been widely debated [13]. Rather than identifying poor research methodology, assessment tends to identify areas of inadequacy in reporting.

The papers were assessed for quality and we found that most were generally of good quality. However, there were areas where reporting was often poor. These areas were in clearly describing the role of the researcher, and in describing the sampling methodology, as shown in Table 2 below.

**Table 2:** Quality criteria and results

Question	Yes	No	Unclear
Is this study qualitative research?	28	0	0
Are the research questions clearly stated?	28	0	0
Is the qualitative approach clearly justified?	24	4	0
Is the approach appropriate for the research question?	24	0	4
Is the study context clearly described?	28	0	0
Is the role of the researcher clearly described?	13	15	0
Is the sampling method clearly described?	14	14	0
Is the sampling strategy appropriate for the research question?	14	0	14
Is the method of data collection clearly described?	25	2	1
Is the data collection method appropriate to the research question?	25	0	3
Is the method of analysis clearly described?	22	4	2
Is the analysis appropriate for the research question?	23	0	5
Are the claims made supported by sufficient evidence?	28	0	0

Authors of the papers which did not report data required for the quality assessment were not contacted to try and obtain the information. This decision was made because we were not excluding papers based on quality and the details were not required for the analysis we were conducting.

#### Barriers to service use by ethnic minorities

The original themes presented by the authors of the papers were grouped and reinterpreted to develop a number of new concepts around barriers to accessing dementia care (available in Additional file 2). Further consideration and discussion by the authors divided the new concepts into two new overarching concepts: inadequacies and *cultural habitus*/experience (Table 3).

**Table 3:** Barriers and facilitators

	BARRIERS	FACILITATORS
Overarching concept	New concept	New concept
Inadequacies	Lack of carer/patient understanding of the causes and symptoms of dementia	Improving knowledge of dementia
	Lack of Knowledge, familiarity and awareness of services and how to navigate the system	
	Professionals lack of specialist knowledge and lack of cultural and linguistic ability to achieve diagnosis	Improving training for professionals in identifying and screening for dementia
	Limitations of available services and lack of cultural awareness	Broadening and adapting servicesContinuity of care
	Preconceived ideas of treatability /normalisation	
Cultural habitus/experiences	Anxiety/trust issues about ‘outside’ support and its cultural appropriateness	Tackling the issue of ‘outsiders’
	Issues around trust and racism, both historic and current	
	Societal stigma of mental illness and denial or concealment as a defence against the reactions of others	Addressing societal stigma/denial/concealment
	Cultural issues impacting on perceptions of Western medicine and the acceptability of services	
	The impact of cultural/familial expectations and community perceptions on care decisions	
	Health care provider exclusion and dismissal of carer concerns/Negative carer experiences of help seeking	
	Negative emotions associated with response from own community	

##### Inadequacies

The first overarching concept was of inadequacies, this incorporated all of the original themes around deficits in education and service provision. It was often reported (*n* = 19) that both general community members and carers for people with dementia felt that they did not have the necessary information about dementia – its causes, its symptoms and prognosis.

“they had little knowledge of the condition of dementia or how quality of life might be improved”. #6, pp388

“Families described their own misperceptions and lack of understanding about the nature of dementia as the main barriers to early diagnosis”. #11, pp165

Many of the studies also reported that the lack of knowledge of the disease and of its causes and symptoms, led in many cases to normalisation of dementia as part of getting older. Linked to this concept were preconceived ideas on the treatability of dementia with many reporting that as family carers did not view dementia as a disease it was therefore not treatable and so the perceived need to seek medical intervention was absent.

“families assumed that the dementia symptoms were natural for an older person and, thus, denied the need to seek medical attention or other resources”. #5, pp118

"None of the participants viewed mind loss as a reason to seek health care”. #13, pp7

“it was not clear to them why going to a doctor would be helpful, calling it “a waste of time,” stating, “the doctor can’t do much to bring my father’s memory back”, or they attributed the changes to normal aging, “my mother is old. ”” #19, pp8

As well as lack of knowledge about the disease, many papers (*n* = 12) also reported a lack of knowledge, familiarity and awareness of services available to dementia patients. This was further compounded by an inability to successfully navigate the system with language barriers being cited as a major obstacle to accessing relevant information.

“reflected that they had been bewildered about the availability of services and how they could be accessed”. #18, pp244

“The biggest concern expressed by participants was that mainstream dementia services did not often have bilingual staff”. #24, pp5

The lack of knowledge was not limited to carers or the community but also within the health care system. Several studies (*n* = 9) reported on the lack of specialist dementia knowledge, particularly among GPs. In many cases the attitudes of GPs were seen to reinforce normalisation of memory loss as part of ageing, resulting in minimisation of carer concerns and failure to reach diagnosis.

“that GP time was limited and GPs would prioritise severe dementia and physical illnesses and dismiss memory problems as being due to old age”. #30, pp5

“newly knowledgeable family members encountered medical providers who were less well informed about dementia. Lack of specialized knowledge on the part of medical providers in some cases interfered with initial diagnosis” #11.pp166

There were also a number of other inadequacies in the healthcare system that were highlighted by the studies, such as service limitations (*n* = 7). Those that did, reported on the lack of choice carers and patients face when seeking formal support services. Services often lacked cultural awareness and diversity for interacting with different cultural communities. Those services that did cater to the needs of minority patients were often geographically widely dispersed with long waiting lists, resulting in poor accessibility for carers and patients.

“The main problems identified with ethno-specific services were the long waiting lists due to the high demand, the observation that staff were not always well trained in dementia and that some staff did not speak the language of the community that the service was catering to”. #24, pp5

“Some participants also felt that services needed to have a better understanding of cultural needs and the lack of it led to worse outcomes”. #30, pp5

“general shortage of dementia services across the region, never mind the dearth of specialist provision for marginalised groups”. #10, pp71

##### Cultural habitus

Cultural habitus was a concept devised by Bourdieu to refer to socialised norms or tendencies that guide behaviour and thinking. It describes the deeply ingrained habits, skills, and dispositions people exhibit as a result of life experience [14]. Cultural issues were often seen to play a major role in families seeking help with dementia patients.

Many of the barriers to accessing care described in the papers reflected the strong impact of cultural norms within ethnic minority groups. This was seen to have a huge impact on care decisions and perceptions of Western medicine and service access.

“They also share a pervasive mistrust of government assistance and the medical system”. #5, pp117

“Families often reported that the structure and rules of professional caregiving systems impacted significantly on their care decisions”. #2, pp443

Stigma around mental health and dementia was a fairly consistent theme, evident in many of the studies (*n* = 13). As a result of community responses carers and family members displayed feelings of guilt, denial, fear, embarrassment and shame. Denial and concealment of the condition was evident as a result of the associated stigma.

“The presence of a very high degree of stigma associated with it, and with other mental health problems, was revealed, contributing to the reluctance to acknowledge symptoms and get help” #9, pp23

“Shame and stigma are among the most difficult barriers to surmount for many Asian families”. #14, pp20

“Families commonly responded with denial, fear, and embarrassment when their elders exhibited dementia symptoms”.#5, pp118

Of particular importance was the concept of ‘outsiders’ often related to issues of trust and perceptions of racism. Many authors (*n* = 11) reported that interviewees expressed anxiety and reluctance about letting other people come into the home to provide care or support. There is little choice within the health and social care systems and so matching professional carers by language, religion or gender is rare and hard to achieve. This results in care options that are considered unacceptable particularly to certain communities.

“Arabic communities struggled with having an outsider come into the home and some Arabic family carers stated that personal care, even by someone of the same gender, was unacceptable”. #24, pp6

“When health care was received it had not been found acceptable and fears of racism were expressed”. #13, pp8

Community expectations were reported in 10 papers as a barrier to formal services and were seen to put increased pressure on family members as clearly defined responsibilities and obligations to the family were enforced. Such traditions and beliefs are not easily addressed. Cultural impacts were further reinforced by perceptions of institutionalised racism and negative carer experiences of help seeking resulting in further issues around trust in the health and social care systems. Beliefs around western medicine were also identified as a barrier as some authors (*n* = 9) reported strongly held folk beliefs around health, ageing dementia.

“expressed similar notions of having ‘no choice’ but to cope with their carer role which, for them, was due to the expectation to cope with situations of stress and adversity from older members within the Sikh community”. #29, pp1047"

“Believing in curses, punishment, and possession led individuals to seek non-medical cures”. #5, pp123

“perception of negative health care provider attitudes and behavior encountered during the process of obtaining a diagnosis”. #7, pp227

#### Facilitators to service use by ethnic minorities

Few of the papers in this review focussed on facilitators to service use. In fact only four papers focussed on it in a meaningful way (5,6,24,25). As a result there was much less information on facilitators available. Most of the reported facilitators mapped on to service level interventions, so educating patients and carers about dementia, improving screening and recognition of dementia by GPs, and broadening and adapting services to meet specific population needs were seen as key.

“The importance of a sympathetic GP and continuity of care from a professional were also emphasized”. #22, pp1075

“a need to develop awareness of and knowledge about dementia, and to promote more culturally sensitive practice among health and social care professionals”. #6, pp391

“Bilingual workers from ethno-specific or multicultural agencies could provide a personal and tailored service because they had an understanding of the language and culture of the clients and their families”. #24,pp5"

“When planning meals and program activities, services need to provide a range of options that reflect the cultural diversity of service users”. #24, pp8

There was some discussion of cultural issues but there was very limited information presented as to what might reduce the impact on help seeking and accessing care services. The only concepts covered were addressing societal stigma/denial/concealment, mainly through education and outreach within the community; and working with the community to build trust and confidence in care providers.

“outreach materials and interactions needed to be culturally sensitive and tailored to the targeted population”. #5, pp121

“Presenting dementia as a medical condition was widely favoured …the use of a physical illness idiom could help move people towards a more appropriate social model of dementia, without stigma or ‘supernatural’ connotations”. #6, pp392

“normalising help-seeking and breaking down the stigma associated with both the symptoms and help-seeking might encourage earlier help-seeking”. #30, pp6

“Providers found it helpful to offer traditional food, and to celebrate national holidays of their clients’ home countries. Additionally, providers developed service activities around social themes in non-institutional settings. Support groups were referred to as “clubs” or “tea-time.” “ #5, pp126

## Discussion

The review identified 28 qualitative papers around barriers and facilitators to dementia services by ethnic minority populations. We conducted a meta-synthesis that has not previously been undertaken, with the aim of better understanding the relationships between barriers and facilitators to align them with future intervention approaches.

Many of the papers included in the synthesis reported similar themes in terms of perceived barriers to dementia diagnosis and access to care. Whilst identifying these barriers is important, papers tended to focus on these as isolated themes within their study population. This meta synthesis brings together those studies to generate a new understanding of the existing research. Whilst different ethnic minority groups should not be considered as a single homogenous group, there are commonalities that result in reduced understanding of dementia and the importance of diagnosis and treatment.

We developed two overarching concepts from the literature: inadequacies and *cultural habitus*. We suggest that the two concepts lend themselves to interventions at either a service level (inadequacies) or a community level (*cultural habitus*). There was a paucity of research focussed on facilitating factors and most of that research reflected on service level facilitators. To achieve a more sustainable approach to facilitating access to services there is an argument that it is as important to educate ethnic minority communities about how health professionals and health services work as it is vice versa [15].

Inadequacies tended to be linked to service level barriers mainly focussing on the need for education that is inclusive of and directly related to the target population. To tailor the literature and education, simply translating generic versions of patient information is not adequate [16]. There is a need for redesign of the literature making it socially and culturally appropriate, for example, by featuring pictures and case examples from the relevant community.

Dissemination of information is also important. Research suggests current approaches may not have enough penetration into the ethnic groups. Alternates via community organisations, media or community champions, needs to be further explored [17].

Diagnosis of dementia in ethnic minority populations remains problematic due in part to the diagnostic tools and skills of primary care staff and in part to cultural beliefs. As Primary care is often the initial point for recognition and diagnosis of dementia, this is a key area for further research. Diagnosis opens the doorway to access to services and also serves to identify who the patient’s primary carers are and what support they need.

There is an assumption that ethnic minority populations prefer to ‘care for their own’, and whilst the literature supports this to some degree, this should not be relied on as an alternative to formal support and intervention [18]. The literature also shows that as in other cultures the burden placed on family members is great and there often comes a point where it is not possible to give a patient the care they need without professional and formal care arrangements.

Many of the papers focussed on the need for service level interventions, tailoring services to specific ethnic groups. One reason for this may be that it may be an easier area to address than trying to impact changes at a cultural level within target populations. With increased resources and suitable adaptations to processes such as information provision, training for health professionals and culturally adapting services to specific needs of a population, many of these barriers could be addressed, but it would come at significant cost to the health service [19].

There was also some focus on cultural engagement and whilst interacting with the minority communities may help acceptance to some degree, it may not result in substantial changes in beliefs and behaviour. Aspects of cultural habitus can be both overt and covert in nature (people are generally not aware of how it impacts on them), even with investment in services they may not be amenable to change or it would likely be part of a long process which requires years of intervention to produce small changes.

**Fig. 2:**
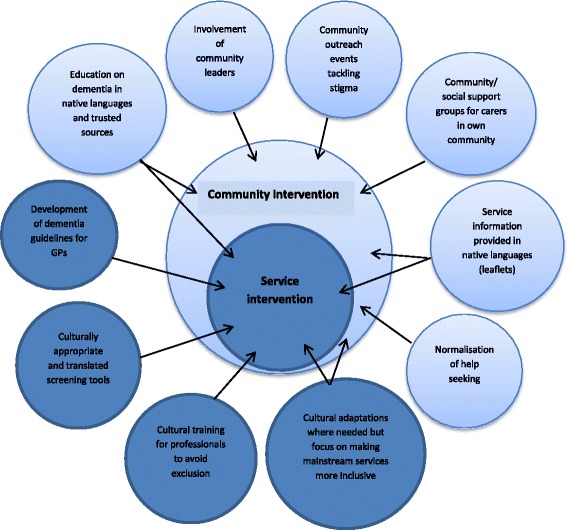
A model for intervention

The two concepts of inadequacies and cultural habitus are strongly linked and in no way independent of each other therefore, to address them separately is unlikely to produce desired results. This has also been explored in other areas such as the AMP model aimed at improving access to psychosocial interventions for common mental health problems [20]. Intervention at a service level, working to improve knowledge of dementia in the population and improving practitioner recognition of the problem and adapting services to give patients more choice will not work if cultural beliefs/expectations are not also taken in to consideration [20]. Our findings suggest that service level interventions could benefit by take place within community based interventions (Fig. 2).

Without parallel interventions a decision is needed about priorities for implementing interventions to improve care for ethnic minority groups. Is it better a) to spend time and resources on culturally adapting services, when without community based intervention, those services are unlikely to be utilised? Or b) first implementing an intervention at a community level, improving understanding and hopefully acceptance of services, but not having suitable adapted services to refer that community to?

The former would create a service which has no customers, and the latter would increase the need for services but would not be able to provide suitably adapted services.

### Future direction

Population and health statistics show that dementia is a key concern and issues around culture will not disappear as the current immigrant population ages as population movement continues. If the cultural preference is to care for their community member with dementia themselves, services need to be adapted to take this in to consideration but not to rely upon it. Ongoing monitoring and support of carers is also important.

We have suggested a model in which intervention should occur at both a service level and a community level at the same time. Further research is required, supported by the processes of implementation science to explore ways in which dual targeted interventions could be implemented in primary care.

#### Strengths and limitations

The decision was made to focus on peer reviewed published work. Therefore we did not search the ‘grey literature’ for this review. Even though a systematic review of the literature was performed, it is possible that there are other studies that were not identified. This may be in part due to the lack of standardisation in terminologies used in this area. We focussed on papers that identified and reported on dementia and Alzheimer’s Disease rather than ‘memory problems’ or ‘memory loss’ as there may be other causes for these conditions. However, we found 28 papers that fit our criteria, giving a large body of work for conducting a meta-ethnographic analysis. While papers had varying approaches and differing samples, there was evidence for data saturation across many themes.

Some of the themes raised are not necessarily specific to ethnic minority populations, for example, normalisation of dementia as part of ageing which can be seen in the general population. However, these themes and the suggested model for intervention still incorporate these barriers. The fact that some barriers are experienced by general populations does not detract from their relevance to ethnic minority groups.

We did not make a comparison of themes between ethnic minority populations or to general populations although this may provide a fruitful avenue for future research.

## Conclusions

This review aimed to identify relevant papers that used qualitative methods to explore barriers and facilitators to accessing dementia care in ethnic minorities. The meta synthesis used a process of re-conceptualisation to further our understanding of barriers and facilitators across ethnic minority groups. Our results suggest that barriers should be addressed on two levels, service level interventions and personal or community level interventions, in conjunction to try and maximise their effectiveness. Further research in terms of the implementation of dual intervention strategies is needed.

## Additional files

Additional file 1: Example search strategy for Ovid MEDLINE(R). Search strategy and search terms used to identify relevant studies. (DOCX 131 kb)

Additional file 2: Table showing the original themes around barriers extracted from papers with recoding to new concepts. Table showing the themes extracted from papers along with how they were ordered and recoded and how they were then grouped under new overarching concepts. (DOCX 20 kb)
