# Supplementary material for: Barriers and facilitators in accessing dementia care by ethnic minority groups: a meta-synthesis of qualitative studies
Source: BMC Psychiatry. 2017 Aug 30;17:316. doi: 10.1186/s12888-017-1474-0 (PMC5577676; doi:10.1186/s12888-017-1474-0)
Supplement: Supplementary file 1 — Example search strategy for Ovid MEDLINE(R). Search strategy and search terms used to identify relevant studies. (DOCX 131 kb) [file 12888_2017_1474_MOESM1_ESM.docx]

**Additional file 1**

**Example search strategy for Ovid MEDLINE(R)**

| \| **** \| [# ▲](http://ovidsp.uk.ovid.com/sp-3.19.0a/ovidweb.cgi?&S=PDPDPDOCEFHFCOKDFNIKHFJHAIAPAA00&Sort+Sets=descending) \| **Searches** \| **Results** \| **Type** \| **Actions** \| **Annotations** \| \| --- \| --- \| --- \| --- \| --- \| --- \| --- \| \|  \| \| \| \| \| \| \| \|  \| 1 \| Dementia/ or dementia.mp. \| 84164 \| Advanced \| [Display Results](http://ovidsp.uk.ovid.com/sp-3.19.0a/ovidweb.cgi?&S=PDPDPDOCEFHFCOKDFNIKHFJHAIAPAA00&SELECT=S.sh%7c&R=1&Process+Action=display)  [More](http://ovidsp.uk.ovid.com/sp-3.19.0a/ovidweb.cgi) \|  \| \|  \| 2 \| Alzheimer Disease/ or Alzheimer* disease.mp. \| 101182 \| Advanced \| [Display Results](http://ovidsp.uk.ovid.com/sp-3.19.0a/ovidweb.cgi?&S=PDPDPDOCEFHFCOKDFNIKHFJHAIAPAA00&SELECT=S.sh%7c&R=2&Process+Action=display)  [More](http://ovidsp.uk.ovid.com/sp-3.19.0a/ovidweb.cgi) \|  \| \|  \| 3 \| 1 or 2 \| 155203 \| Advanced \| [Display Results](http://ovidsp.uk.ovid.com/sp-3.19.0a/ovidweb.cgi?&S=PDPDPDOCEFHFCOKDFNIKHFJHAIAPAA00&SELECT=S.sh%7c&R=3&Process+Action=display)  [More](http://ovidsp.uk.ovid.com/sp-3.19.0a/ovidweb.cgi) \|  \| \|  \| 4 \| ethnic*.mp. \| 126694 \| Advanced \| [Display Results](http://ovidsp.uk.ovid.com/sp-3.19.0a/ovidweb.cgi?&S=PDPDPDOCEFHFCOKDFNIKHFJHAIAPAA00&SELECT=S.sh%7c&R=4&Process+Action=display)  [More](http://ovidsp.uk.ovid.com/sp-3.19.0a/ovidweb.cgi) \|  \| \|  \| 5 \| Asian.mp. or Asian Continental Ancestry Group/ \| 89665 \| Advanced \| [Display Results](http://ovidsp.uk.ovid.com/sp-3.19.0a/ovidweb.cgi?&S=PDPDPDOCEFHFCOKDFNIKHFJHAIAPAA00&SELECT=S.sh%7c&R=5&Process+Action=display)  [More](http://ovidsp.uk.ovid.com/sp-3.19.0a/ovidweb.cgi) \|  \| \|  \| 6 \| black.mp. or African Continental Ancestry Group/ \| 104349 \| Advanced \| [Display Results](http://ovidsp.uk.ovid.com/sp-3.19.0a/ovidweb.cgi?&S=PDPDPDOCEFHFCOKDFNIKHFJHAIAPAA00&SELECT=S.sh%7c&R=6&Process+Action=display)  [More](http://ovidsp.uk.ovid.com/sp-3.19.0a/ovidweb.cgi) \|  \| \|  \| 7 \| Ethnic Groups/ or multiethnic.mp. \| 54225 \| Advanced \| [Display Results](http://ovidsp.uk.ovid.com/sp-3.19.0a/ovidweb.cgi?&S=PDPDPDOCEFHFCOKDFNIKHFJHAIAPAA00&SELECT=S.sh%7c&R=7&Process+Action=display)  [More](http://ovidsp.uk.ovid.com/sp-3.19.0a/ovidweb.cgi) \|  \| \|  \| 8 \| 4 or 5 or 6 or 7 \| 288052 \| Advanced \| [Display Results](http://ovidsp.uk.ovid.com/sp-3.19.0a/ovidweb.cgi?&S=PDPDPDOCEFHFCOKDFNIKHFJHAIAPAA00&SELECT=S.sh%7c&R=8&Process+Action=display)  [More](http://ovidsp.uk.ovid.com/sp-3.19.0a/ovidweb.cgi) \|  \| \|  \| 9 \| 3 and 8 \| 2556 \| Advanced \| [Display Results](http://ovidsp.uk.ovid.com/sp-3.19.0a/ovidweb.cgi?&S=PDPDPDOCEFHFCOKDFNIKHFJHAIAPAA00&SELECT=S.sh%7c&R=9&Process+Action=display)  [More](http://ovidsp.uk.ovid.com/sp-3.19.0a/ovidweb.cgi) \|  \| \|  \| 10 \| Grounded Theory/ \| 204 \| Advanced \| [Display Results](http://ovidsp.uk.ovid.com/sp-3.19.0a/ovidweb.cgi?&S=PDPDPDOCEFHFCOKDFNIKHFJHAIAPAA00&SELECT=S.sh%7c&R=10&Process+Action=display)  [More](http://ovidsp.uk.ovid.com/sp-3.19.0a/ovidweb.cgi) \|  \| \|  \| 11 \| Interview/ or interview*.mp. \| 259417 \| Advanced \| [Display Results](http://ovidsp.uk.ovid.com/sp-3.19.0a/ovidweb.cgi?&S=PDPDPDOCEFHFCOKDFNIKHFJHAIAPAA00&SELECT=S.sh%7c&R=11&Process+Action=display)  [More](http://ovidsp.uk.ovid.com/sp-3.19.0a/ovidweb.cgi) \|  \| \|  \| 12 \| content analysis.mp. \| 12584 \| Advanced \| [Display Results](http://ovidsp.uk.ovid.com/sp-3.19.0a/ovidweb.cgi?&S=PDPDPDOCEFHFCOKDFNIKHFJHAIAPAA00&SELECT=S.sh%7c&R=12&Process+Action=display)  [More](http://ovidsp.uk.ovid.com/sp-3.19.0a/ovidweb.cgi) \|  \| \|  \| 13 \| Focus Groups/ or focus group*.mp. \| 28089 \| Advanced \| [Display Results](http://ovidsp.uk.ovid.com/sp-3.19.0a/ovidweb.cgi?&S=PDPDPDOCEFHFCOKDFNIKHFJHAIAPAA00&SELECT=S.sh%7c&R=13&Process+Action=display)  [More](http://ovidsp.uk.ovid.com/sp-3.19.0a/ovidweb.cgi) \|  \| \|  \| 14 \| discourse*.mp. \| 9320 \| Advanced \| [Display Results](http://ovidsp.uk.ovid.com/sp-3.19.0a/ovidweb.cgi?&S=PDPDPDOCEFHFCOKDFNIKHFJHAIAPAA00&SELECT=S.sh%7c&R=14&Process+Action=display)  [More](http://ovidsp.uk.ovid.com/sp-3.19.0a/ovidweb.cgi) \|  \| \|  \| 15 \| ethnograph*.mp. \| 6318 \| Advanced \| [Display Results](http://ovidsp.uk.ovid.com/sp-3.19.0a/ovidweb.cgi?&S=PDPDPDOCEFHFCOKDFNIKHFJHAIAPAA00&SELECT=S.sh%7c&R=15&Process+Action=display)  [More](http://ovidsp.uk.ovid.com/sp-3.19.0a/ovidweb.cgi) \|  \| \|  \| 16 \| ethnonursing.mp. \| 91 \| Advanced \| [Display Results](http://ovidsp.uk.ovid.com/sp-3.19.0a/ovidweb.cgi?&S=PDPDPDOCEFHFCOKDFNIKHFJHAIAPAA00&SELECT=S.sh%7c&R=16&Process+Action=display)  [More](http://ovidsp.uk.ovid.com/sp-3.19.0a/ovidweb.cgi) \|  \| \|  \| 17 \| phenomenological*.mp. \| 9732 \| Advanced \| [Display Results](http://ovidsp.uk.ovid.com/sp-3.19.0a/ovidweb.cgi?&S=PDPDPDOCEFHFCOKDFNIKHFJHAIAPAA00&SELECT=S.sh%7c&R=17&Process+Action=display)  [More](http://ovidsp.uk.ovid.com/sp-3.19.0a/ovidweb.cgi) \|  \| \|  \| 18 \| qualitative*.mp. or Qualitative Research/ \| 164808 \| Advanced \| [Display Results](http://ovidsp.uk.ovid.com/sp-3.19.0a/ovidweb.cgi?&S=PDPDPDOCEFHFCOKDFNIKHFJHAIAPAA00&SELECT=S.sh%7c&R=18&Process+Action=display)  [More](http://ovidsp.uk.ovid.com/sp-3.19.0a/ovidweb.cgi) \|  \| \|  \| 19 \| 10 or 11 or 12 or 13 or 14 or 15 or 16 or 17 or 18 \| 412967 \| Advanced \| [Display Results](http://ovidsp.uk.ovid.com/sp-3.19.0a/ovidweb.cgi?&S=PDPDPDOCEFHFCOKDFNIKHFJHAIAPAA00&SELECT=S.sh%7c&R=19&Process+Action=display)  [More](http://ovidsp.uk.ovid.com/sp-3.19.0a/ovidweb.cgi) \|  \| \|  \| 20 \| 9 and 19 \| 259 \| Advanced \|  \|  \| |
| --- | --- | --- | --- | --- | --- | --- | --- | --- | --- | --- | --- | --- | --- | --- | --- | --- | --- | --- | --- | --- | --- | --- | --- | --- | --- | --- | --- | --- | --- | --- | --- | --- | --- | --- | --- | --- | --- | --- | --- | --- | --- | --- | --- | --- | --- | --- | --- | --- | --- | --- | --- | --- | --- | --- | --- | --- | --- | --- | --- | --- | --- | --- | --- | --- | --- | --- | --- | --- | --- | --- | --- | --- | --- | --- | --- | --- | --- | --- | --- | --- | --- | --- | --- | --- | --- | --- | --- | --- | --- | --- | --- | --- | --- | --- | --- | --- | --- | --- | --- | --- | --- | --- | --- | --- | --- | --- | --- | --- | --- | --- | --- | --- | --- | --- | --- | --- | --- | --- | --- | --- | --- | --- | --- | --- | --- | --- | --- | --- | --- | --- | --- | --- | --- | --- | --- | --- | --- | --- | --- | --- | --- | --- | --- | --- | --- | --- | --- | --- | --- | --- | --- | --- | --- | --- |

**PsycINFO 1806 to April Week 3 2016**

| \| **** \| [# ▲](http://ovidsp.uk.ovid.com/sp-3.19.0a/ovidweb.cgi?&S=PDPDPDOCEFHFCOKDFNIKHFJHAIAPAA00&Sort+Sets=descending) \| **Searches** \| **Results** \| **Type** \| **Actions** \| **Annotations** \| \| --- \| --- \| --- \| --- \| --- \| --- \| --- \| \|  \| \| \| \| \| \| \| \|  \| 1 \| Dementia/ or dementia.mp. \| 55984 \| Advanced \| [Display Results](http://ovidsp.uk.ovid.com/sp-3.19.0a/ovidweb.cgi?&S=PDPDPDOCEFHFCOKDFNIKHFJHAIAPAA00&SELECT=S.sh%7c&R=1&Process+Action=display)  [More](http://ovidsp.uk.ovid.com/sp-3.19.0a/ovidweb.cgi?&S=PDPDPDOCEFHFCOKDFNIKHFJHAIAPAA00&SELECT=S.sh%7c&Expand=1&Main+Search+Page=Main+Search+Page) \|  \| \|  \| 2 \| Alzheimer Disease/ or Alzheimer* disease.mp. \| 47206 \| Advanced \| [Display Results](http://ovidsp.uk.ovid.com/sp-3.19.0a/ovidweb.cgi?&S=PDPDPDOCEFHFCOKDFNIKHFJHAIAPAA00&SELECT=S.sh%7c&R=2&Process+Action=display)  [More](http://ovidsp.uk.ovid.com/sp-3.19.0a/ovidweb.cgi?&S=PDPDPDOCEFHFCOKDFNIKHFJHAIAPAA00&SELECT=S.sh%7c&Expand=1&Main+Search+Page=Main+Search+Page) \|  \| \|  \| 3 \| 1 or 2 \| 80960 \| Advanced \| [Display Results](http://ovidsp.uk.ovid.com/sp-3.19.0a/ovidweb.cgi?&S=PDPDPDOCEFHFCOKDFNIKHFJHAIAPAA00&SELECT=S.sh%7c&R=3&Process+Action=display)  [More](http://ovidsp.uk.ovid.com/sp-3.19.0a/ovidweb.cgi?&S=PDPDPDOCEFHFCOKDFNIKHFJHAIAPAA00&SELECT=S.sh%7c&Expand=1&Main+Search+Page=Main+Search+Page) \|  \| \|  \| 4 \| ethnic*.mp. \| 104058 \| Advanced \| [Display Results](http://ovidsp.uk.ovid.com/sp-3.19.0a/ovidweb.cgi?&S=PDPDPDOCEFHFCOKDFNIKHFJHAIAPAA00&SELECT=S.sh%7c&R=4&Process+Action=display)  [More](http://ovidsp.uk.ovid.com/sp-3.19.0a/ovidweb.cgi?&S=PDPDPDOCEFHFCOKDFNIKHFJHAIAPAA00&SELECT=S.sh%7c&Expand=1&Main+Search+Page=Main+Search+Page) \|  \| \|  \| 5 \| Asian.mp. or Asian Continental Ancestry Group/ \| 21608 \| Advanced \| [Display Results](http://ovidsp.uk.ovid.com/sp-3.19.0a/ovidweb.cgi?&S=PDPDPDOCEFHFCOKDFNIKHFJHAIAPAA00&SELECT=S.sh%7c&R=5&Process+Action=display)  [More](http://ovidsp.uk.ovid.com/sp-3.19.0a/ovidweb.cgi?&S=PDPDPDOCEFHFCOKDFNIKHFJHAIAPAA00&SELECT=S.sh%7c&Expand=1&Main+Search+Page=Main+Search+Page) \|  \| \|  \| 6 \| black.mp. or African Continental Ancestry Group/ \| 49296 \| Advanced \| [Display Results](http://ovidsp.uk.ovid.com/sp-3.19.0a/ovidweb.cgi?&S=PDPDPDOCEFHFCOKDFNIKHFJHAIAPAA00&SELECT=S.sh%7c&R=6&Process+Action=display)  [More](http://ovidsp.uk.ovid.com/sp-3.19.0a/ovidweb.cgi?&S=PDPDPDOCEFHFCOKDFNIKHFJHAIAPAA00&SELECT=S.sh%7c&Expand=1&Main+Search+Page=Main+Search+Page) \|  \| \|  \| 7 \| Ethnic Groups/ or multiethnic.mp. \| 12610 \| Advanced \| [Display Results](http://ovidsp.uk.ovid.com/sp-3.19.0a/ovidweb.cgi?&S=PDPDPDOCEFHFCOKDFNIKHFJHAIAPAA00&SELECT=S.sh%7c&R=7&Process+Action=display)  [More](http://ovidsp.uk.ovid.com/sp-3.19.0a/ovidweb.cgi?&S=PDPDPDOCEFHFCOKDFNIKHFJHAIAPAA00&SELECT=S.sh%7c&Expand=1&Main+Search+Page=Main+Search+Page) \|  \| \|  \| 8 \| 4 or 5 or 6 or 7 \| 148307 \| Advanced \| [Display Results](http://ovidsp.uk.ovid.com/sp-3.19.0a/ovidweb.cgi?&S=PDPDPDOCEFHFCOKDFNIKHFJHAIAPAA00&SELECT=S.sh%7c&R=8&Process+Action=display)  [More](http://ovidsp.uk.ovid.com/sp-3.19.0a/ovidweb.cgi?&S=PDPDPDOCEFHFCOKDFNIKHFJHAIAPAA00&SELECT=S.sh%7c&Expand=1&Main+Search+Page=Main+Search+Page) \|  \| \|  \| 9 \| 3 and 8 \| 1672 \| Advanced \| [Display Results](http://ovidsp.uk.ovid.com/sp-3.19.0a/ovidweb.cgi?&S=PDPDPDOCEFHFCOKDFNIKHFJHAIAPAA00&SELECT=S.sh%7c&R=9&Process+Action=display)  [More](http://ovidsp.uk.ovid.com/sp-3.19.0a/ovidweb.cgi?&S=PDPDPDOCEFHFCOKDFNIKHFJHAIAPAA00&SELECT=S.sh%7c&Expand=1&Main+Search+Page=Main+Search+Page) \|  \| \|  \| 10 \| Grounded Theory/ \| 3042 \| Advanced \| [Display Results](http://ovidsp.uk.ovid.com/sp-3.19.0a/ovidweb.cgi?&S=PDPDPDOCEFHFCOKDFNIKHFJHAIAPAA00&SELECT=S.sh%7c&R=10&Process+Action=display)  [More](http://ovidsp.uk.ovid.com/sp-3.19.0a/ovidweb.cgi?&S=PDPDPDOCEFHFCOKDFNIKHFJHAIAPAA00&SELECT=S.sh%7c&Expand=1&Main+Search+Page=Main+Search+Page) \|  \| \|  \| 11 \| Interview/ or interview*.mp. \| 304176 \| Advanced \| [Display Results](http://ovidsp.uk.ovid.com/sp-3.19.0a/ovidweb.cgi?&S=PDPDPDOCEFHFCOKDFNIKHFJHAIAPAA00&SELECT=S.sh%7c&R=11&Process+Action=display)  [More](http://ovidsp.uk.ovid.com/sp-3.19.0a/ovidweb.cgi?&S=PDPDPDOCEFHFCOKDFNIKHFJHAIAPAA00&SELECT=S.sh%7c&Expand=1&Main+Search+Page=Main+Search+Page) \|  \| \|  \| 12 \| content analysis.mp. \| 19435 \| Advanced \| [Display Results](http://ovidsp.uk.ovid.com/sp-3.19.0a/ovidweb.cgi?&S=PDPDPDOCEFHFCOKDFNIKHFJHAIAPAA00&SELECT=S.sh%7c&R=12&Process+Action=display)  [More](http://ovidsp.uk.ovid.com/sp-3.19.0a/ovidweb.cgi?&S=PDPDPDOCEFHFCOKDFNIKHFJHAIAPAA00&SELECT=S.sh%7c&Expand=1&Main+Search+Page=Main+Search+Page) \|  \| \|  \| 13 \| Focus Groups/ or focus group*.mp. \| 24823 \| Advanced \| [Display Results](http://ovidsp.uk.ovid.com/sp-3.19.0a/ovidweb.cgi?&S=PDPDPDOCEFHFCOKDFNIKHFJHAIAPAA00&SELECT=S.sh%7c&R=13&Process+Action=display)  [More](http://ovidsp.uk.ovid.com/sp-3.19.0a/ovidweb.cgi?&S=PDPDPDOCEFHFCOKDFNIKHFJHAIAPAA00&SELECT=S.sh%7c&Expand=1&Main+Search+Page=Main+Search+Page) \|  \| \|  \| 14 \| discourse*.mp. \| 44706 \| Advanced \| [Display Results](http://ovidsp.uk.ovid.com/sp-3.19.0a/ovidweb.cgi?&S=PDPDPDOCEFHFCOKDFNIKHFJHAIAPAA00&SELECT=S.sh%7c&R=14&Process+Action=display)  [More](http://ovidsp.uk.ovid.com/sp-3.19.0a/ovidweb.cgi?&S=PDPDPDOCEFHFCOKDFNIKHFJHAIAPAA00&SELECT=S.sh%7c&Expand=1&Main+Search+Page=Main+Search+Page) \|  \| \|  \| 15 \| ethnograph*.mp. \| 22179 \| Advanced \| [Display Results](http://ovidsp.uk.ovid.com/sp-3.19.0a/ovidweb.cgi?&S=PDPDPDOCEFHFCOKDFNIKHFJHAIAPAA00&SELECT=S.sh%7c&R=15&Process+Action=display)  [More](http://ovidsp.uk.ovid.com/sp-3.19.0a/ovidweb.cgi?&S=PDPDPDOCEFHFCOKDFNIKHFJHAIAPAA00&SELECT=S.sh%7c&Expand=1&Main+Search+Page=Main+Search+Page) \|  \| \|  \| 16 \| ethnonursing.mp. \| 54 \| Advanced \| [Display Results](http://ovidsp.uk.ovid.com/sp-3.19.0a/ovidweb.cgi?&S=PDPDPDOCEFHFCOKDFNIKHFJHAIAPAA00&SELECT=S.sh%7c&R=16&Process+Action=display)  [More](http://ovidsp.uk.ovid.com/sp-3.19.0a/ovidweb.cgi?&S=PDPDPDOCEFHFCOKDFNIKHFJHAIAPAA00&SELECT=S.sh%7c&Expand=1&Main+Search+Page=Main+Search+Page) \|  \| \|  \| 17 \| phenomenological*.mp. \| 22395 \| Advanced \| [Display Results](http://ovidsp.uk.ovid.com/sp-3.19.0a/ovidweb.cgi?&S=PDPDPDOCEFHFCOKDFNIKHFJHAIAPAA00&SELECT=S.sh%7c&R=17&Process+Action=display)  [More](http://ovidsp.uk.ovid.com/sp-3.19.0a/ovidweb.cgi?&S=PDPDPDOCEFHFCOKDFNIKHFJHAIAPAA00&SELECT=S.sh%7c&Expand=1&Main+Search+Page=Main+Search+Page) \|  \| \|  \| 18 \| qualitative*.mp. or Qualitative Research/ \| 122232 \| Advanced \| [Display Results](http://ovidsp.uk.ovid.com/sp-3.19.0a/ovidweb.cgi?&S=PDPDPDOCEFHFCOKDFNIKHFJHAIAPAA00&SELECT=S.sh%7c&R=18&Process+Action=display)  [More](http://ovidsp.uk.ovid.com/sp-3.19.0a/ovidweb.cgi?&S=PDPDPDOCEFHFCOKDFNIKHFJHAIAPAA00&SELECT=S.sh%7c&Expand=1&Main+Search+Page=Main+Search+Page) \|  \| \|  \| 19 \| 10 or 11 or 12 or 13 or 14 or 15 or 16 or 17 or 18 \| 448732 \| Advanced \| [Display Results](http://ovidsp.uk.ovid.com/sp-3.19.0a/ovidweb.cgi?&S=PDPDPDOCEFHFCOKDFNIKHFJHAIAPAA00&SELECT=S.sh%7c&R=19&Process+Action=display)  [More](http://ovidsp.uk.ovid.com/sp-3.19.0a/ovidweb.cgi?&S=PDPDPDOCEFHFCOKDFNIKHFJHAIAPAA00&SELECT=S.sh%7c&Expand=1&Main+Search+Page=Main+Search+Page) \|  \| \|  \| 20 \| 9 and 19 \| 280 \| Advance \|  \|  \| |
| --- | --- | --- | --- | --- | --- | --- | --- | --- | --- | --- | --- | --- | --- | --- | --- | --- | --- | --- | --- | --- | --- | --- | --- | --- | --- | --- | --- | --- | --- | --- | --- | --- | --- | --- | --- | --- | --- | --- | --- | --- | --- | --- | --- | --- | --- | --- | --- | --- | --- | --- | --- | --- | --- | --- | --- | --- | --- | --- | --- | --- | --- | --- | --- | --- | --- | --- | --- | --- | --- | --- | --- | --- | --- | --- | --- | --- | --- | --- | --- | --- | --- | --- | --- | --- | --- | --- | --- | --- | --- | --- | --- | --- | --- | --- | --- | --- | --- | --- | --- | --- | --- | --- | --- | --- | --- | --- | --- | --- | --- | --- | --- | --- | --- | --- | --- | --- | --- | --- | --- | --- | --- | --- | --- | --- | --- | --- | --- | --- | --- | --- | --- | --- | --- | --- | --- | --- | --- | --- | --- | --- | --- | --- | --- | --- | --- | --- | --- | --- | --- | --- | --- | --- | --- | --- |

**Embase 1980 to 2016 Week 1**

| \| **** \| [# ▲](http://ovidsp.uk.ovid.com/sp-3.19.0a/ovidweb.cgi?&S=CGHBPDIOHLHFAOPAFNIKOEEGGLDHAA00&Sort+Sets=descending) \| **Searches** \| **Results** \| **Type** \| **Actions** \| **Annotations** \| \| --- \| --- \| --- \| --- \| --- \| --- \| --- \| \|  \| \| \| \| \| \| \| \|  \| 1 \| Dementia/ or dementia.mp. \| 142767 \| Advanced \| [Display Results](http://ovidsp.uk.ovid.com/sp-3.19.0a/ovidweb.cgi?&S=CGHBPDIOHLHFAOPAFNIKOEEGGLDHAA00&SELECT=S.sh%7c&R=1&Process+Action=display)  [More](http://ovidsp.uk.ovid.com/sp-3.19.0a/ovidweb.cgi) \|  \| \|  \| 2 \| Alzheimer Disease/ or Alzheimer* disease.mp. \| 168892 \| Advanced \| [Display Results](http://ovidsp.uk.ovid.com/sp-3.19.0a/ovidweb.cgi?&S=CGHBPDIOHLHFAOPAFNIKOEEGGLDHAA00&SELECT=S.sh%7c&R=2&Process+Action=display)  [More](http://ovidsp.uk.ovid.com/sp-3.19.0a/ovidweb.cgi) \|  \| \|  \| 3 \| 1 or 2 \| 256793 \| Advanced \| [Display Results](http://ovidsp.uk.ovid.com/sp-3.19.0a/ovidweb.cgi?&S=CGHBPDIOHLHFAOPAFNIKOEEGGLDHAA00&SELECT=S.sh%7c&R=3&Process+Action=display)  [More](http://ovidsp.uk.ovid.com/sp-3.19.0a/ovidweb.cgi) \|  \| \|  \| 4 \| ethnic*.mp. \| 211992 \| Advanced \| [Display Results](http://ovidsp.uk.ovid.com/sp-3.19.0a/ovidweb.cgi?&S=CGHBPDIOHLHFAOPAFNIKOEEGGLDHAA00&SELECT=S.sh%7c&R=4&Process+Action=display)  [More](http://ovidsp.uk.ovid.com/sp-3.19.0a/ovidweb.cgi) \|  \| \|  \| 5 \| Asian.mp. or Asian Continental Ancestry Group/ \| 134133 \| Advanced \| [Display Results](http://ovidsp.uk.ovid.com/sp-3.19.0a/ovidweb.cgi?&S=CGHBPDIOHLHFAOPAFNIKOEEGGLDHAA00&SELECT=S.sh%7c&R=5&Process+Action=display)  [More](http://ovidsp.uk.ovid.com/sp-3.19.0a/ovidweb.cgi) \|  \| \|  \| 6 \| black.mp. or African Continental Ancestry Group/ \| 117189 \| Advanced \| [Display Results](http://ovidsp.uk.ovid.com/sp-3.19.0a/ovidweb.cgi?&S=CGHBPDIOHLHFAOPAFNIKOEEGGLDHAA00&SELECT=S.sh%7c&R=6&Process+Action=display)  [More](http://ovidsp.uk.ovid.com/sp-3.19.0a/ovidweb.cgi) \|  \| \|  \| 7 \| Ethnic Groups/ or multiethnic.mp. \| 55284 \| Advanced \| [Display Results](http://ovidsp.uk.ovid.com/sp-3.19.0a/ovidweb.cgi?&S=CGHBPDIOHLHFAOPAFNIKOEEGGLDHAA00&SELECT=S.sh%7c&R=7&Process+Action=display)  [More](http://ovidsp.uk.ovid.com/sp-3.19.0a/ovidweb.cgi) \|  \| \|  \| 8 \| 4 or 5 or 6 or 7 \| 418628 \| Advanced \| [Display Results](http://ovidsp.uk.ovid.com/sp-3.19.0a/ovidweb.cgi?&S=CGHBPDIOHLHFAOPAFNIKOEEGGLDHAA00&SELECT=S.sh%7c&R=8&Process+Action=display)  [More](http://ovidsp.uk.ovid.com/sp-3.19.0a/ovidweb.cgi) \|  \| \|  \| 9 \| 3 and 8 \| 4154 \| Advanced \| [Display Results](http://ovidsp.uk.ovid.com/sp-3.19.0a/ovidweb.cgi?&S=CGHBPDIOHLHFAOPAFNIKOEEGGLDHAA00&SELECT=S.sh%7c&R=9&Process+Action=display)  [More](http://ovidsp.uk.ovid.com/sp-3.19.0a/ovidweb.cgi) \|  \| \|  \| 10 \| Grounded Theory/ \| 3100 \| Advanced \| [Display Results](http://ovidsp.uk.ovid.com/sp-3.19.0a/ovidweb.cgi?&S=CGHBPDIOHLHFAOPAFNIKOEEGGLDHAA00&SELECT=S.sh%7c&R=10&Process+Action=display)  [More](http://ovidsp.uk.ovid.com/sp-3.19.0a/ovidweb.cgi) \|  \| \|  \| 11 \| Interview/ or interview*.mp. \| 348768 \| Advanced \| [Display Results](http://ovidsp.uk.ovid.com/sp-3.19.0a/ovidweb.cgi?&S=CGHBPDIOHLHFAOPAFNIKOEEGGLDHAA00&SELECT=S.sh%7c&R=11&Process+Action=display)  [More](http://ovidsp.uk.ovid.com/sp-3.19.0a/ovidweb.cgi) \|  \| \|  \| 12 \| content analysis.mp. \| 18938 \| Advanced \| [Display Results](http://ovidsp.uk.ovid.com/sp-3.19.0a/ovidweb.cgi?&S=CGHBPDIOHLHFAOPAFNIKOEEGGLDHAA00&SELECT=S.sh%7c&R=12&Process+Action=display)  [More](http://ovidsp.uk.ovid.com/sp-3.19.0a/ovidweb.cgi) \|  \| \|  \| 13 \| Focus Groups/ or focus group*.mp. \| 165200 \| Advanced \| [Display Results](http://ovidsp.uk.ovid.com/sp-3.19.0a/ovidweb.cgi?&S=CGHBPDIOHLHFAOPAFNIKOEEGGLDHAA00&SELECT=S.sh%7c&R=13&Process+Action=display)  [More](http://ovidsp.uk.ovid.com/sp-3.19.0a/ovidweb.cgi) \|  \| \|  \| 14 \| discourse*.mp. \| 12391 \| Advanced \| [Display Results](http://ovidsp.uk.ovid.com/sp-3.19.0a/ovidweb.cgi?&S=CGHBPDIOHLHFAOPAFNIKOEEGGLDHAA00&SELECT=S.sh%7c&R=14&Process+Action=display)  [More](http://ovidsp.uk.ovid.com/sp-3.19.0a/ovidweb.cgi) \|  \| \|  \| 15 \| ethnograph*.mp. \| 8541 \| Advanced \| [Display Results](http://ovidsp.uk.ovid.com/sp-3.19.0a/ovidweb.cgi?&S=CGHBPDIOHLHFAOPAFNIKOEEGGLDHAA00&SELECT=S.sh%7c&R=15&Process+Action=display)  [More](http://ovidsp.uk.ovid.com/sp-3.19.0a/ovidweb.cgi) \|  \| \|  \| 16 \| ethnonursing.mp. \| 96 \| Advanced \| [Display Results](http://ovidsp.uk.ovid.com/sp-3.19.0a/ovidweb.cgi?&S=CGHBPDIOHLHFAOPAFNIKOEEGGLDHAA00&SELECT=S.sh%7c&R=16&Process+Action=display)  [More](http://ovidsp.uk.ovid.com/sp-3.19.0a/ovidweb.cgi) \|  \| \|  \| 17 \| phenomenological*.mp. \| 13663 \| Advanced \| [Display Results](http://ovidsp.uk.ovid.com/sp-3.19.0a/ovidweb.cgi?&S=CGHBPDIOHLHFAOPAFNIKOEEGGLDHAA00&SELECT=S.sh%7c&R=17&Process+Action=display)  [More](http://ovidsp.uk.ovid.com/sp-3.19.0a/ovidweb.cgi) \|  \| \|  \| 18 \| qualitative*.mp. or Qualitative Research/ \| 239764 \| Advanced \| [Display Results](http://ovidsp.uk.ovid.com/sp-3.19.0a/ovidweb.cgi?&S=CGHBPDIOHLHFAOPAFNIKOEEGGLDHAA00&SELECT=S.sh%7c&R=18&Process+Action=display)  [More](http://ovidsp.uk.ovid.com/sp-3.19.0a/ovidweb.cgi) \|  \| \|  \| 19 \| 10 or 11 or 12 or 13 or 14 or 15 or 16 or 17 or 18 \| 693003 \| Advanced \| [Display Results](http://ovidsp.uk.ovid.com/sp-3.19.0a/ovidweb.cgi?&S=CGHBPDIOHLHFAOPAFNIKOEEGGLDHAA00&SELECT=S.sh%7c&R=19&Process+Action=display)  [More](http://ovidsp.uk.ovid.com/sp-3.19.0a/ovidweb.cgi) \|  \| \|  \| 20 \| 9 and 19 \| 372 \| Advanced \| [Display Results](http://ovidsp.uk.ovid.com/sp-3.19.0a/ovidweb.cgi?&S=CGHBPDIOHLHFAOPAFNIKOEEGGLDHAA00&SELECT=S.sh%7c&R=20&Process+Action=display) \|  \| |
| --- | --- | --- | --- | --- | --- | --- | --- | --- | --- | --- | --- | --- | --- | --- | --- | --- | --- | --- | --- | --- | --- | --- | --- | --- | --- | --- | --- | --- | --- | --- | --- | --- | --- | --- | --- | --- | --- | --- | --- | --- | --- | --- | --- | --- | --- | --- | --- | --- | --- | --- | --- | --- | --- | --- | --- | --- | --- | --- | --- | --- | --- | --- | --- | --- | --- | --- | --- | --- | --- | --- | --- | --- | --- | --- | --- | --- | --- | --- | --- | --- | --- | --- | --- | --- | --- | --- | --- | --- | --- | --- | --- | --- | --- | --- | --- | --- | --- | --- | --- | --- | --- | --- | --- | --- | --- | --- | --- | --- | --- | --- | --- | --- | --- | --- | --- | --- | --- | --- | --- | --- | --- | --- | --- | --- | --- | --- | --- | --- | --- | --- | --- | --- | --- | --- | --- | --- | --- | --- | --- | --- | --- | --- | --- | --- | --- | --- | --- | --- | --- | --- | --- | --- | --- | --- |

Bottom of Form

**EBM Reviews - Cochrane Database of Systematic Reviews**2005 to April 20, 2016**,**[
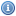
](http://ovidsp.uk.ovid.com/sp-3.19.0a/ovidweb.cgi?&S=CGHBPDIOHLHFAOPAFNIKOEEGGLDHAA00&Database+Field+Guide=3)**EBM Reviews - ACP Journal Club**1991 to April 2016**,**[
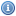
](http://ovidsp.uk.ovid.com/sp-3.19.0a/ovidweb.cgi?&S=CGHBPDIOHLHFAOPAFNIKOEEGGLDHAA00&Database+Field+Guide=7)**EBM Reviews - Database of Abstracts of Reviews of Effects**1st Quarter 2016**,**[
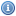
](http://ovidsp.uk.ovid.com/sp-3.19.0a/ovidweb.cgi?&S=CGHBPDIOHLHFAOPAFNIKOEEGGLDHAA00&Database+Field+Guide=4)**EBM Reviews - Cochrane Central Register of Controlled Trials**March 2016**,**[
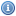
](http://ovidsp.uk.ovid.com/sp-3.19.0a/ovidweb.cgi?&S=CGHBPDIOHLHFAOPAFNIKOEEGGLDHAA00&Database+Field+Guide=6)**EBM Reviews - Cochrane Methodology Register**3rd Quarter 2012**,**[
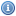
](http://ovidsp.uk.ovid.com/sp-3.19.0a/ovidweb.cgi?&S=CGHBPDIOHLHFAOPAFNIKOEEGGLDHAA00&Database+Field+Guide=8)**EBM Reviews - Health Technology Assessment**1st Quarter 2016**,**[
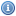
](http://ovidsp.uk.ovid.com/sp-3.19.0a/ovidweb.cgi?&S=CGHBPDIOHLHFAOPAFNIKOEEGGLDHAA00&Database+Field+Guide=9)**EBM Reviews - NHS Economic Evaluation Database**1st Quarter 2016

| [# ▲](http://ovidsp.uk.ovid.com/sp-3.19.0a/ovidweb.cgi?&S=CGHBPDIOHLHFAOPAFNIKOEEGGLDHAA00&Sort+Sets=descending) | **Searches** | **Results** | **Type** | **Actions** | **Annotations** |  |
| --- | --- | --- | --- | --- | --- | --- |
|  | | | | | | |
|  | 1 | Dementia/ or dementia.mp. | 6593 | Advanced | [Display Results](http://ovidsp.uk.ovid.com/sp-3.19.0a/ovidweb.cgi?&S=CGHBPDIOHLHFAOPAFNIKOEEGGLDHAA00&SELECT=S.sh%7c&R=1&Process+Action=display)  [More](http://ovidsp.uk.ovid.com/sp-3.19.0a/ovidweb.cgi?&S=CGHBPDIOHLHFAOPAFNIKOEEGGLDHAA00&SELECT=S.sh%7c&Expand=1&Main+Search+Page=Main+Search+Page) |  |
|  | 2 | Alzheimer Disease/ or Alzheimer* disease.mp. | 5728 | Advanced | [Display Results](http://ovidsp.uk.ovid.com/sp-3.19.0a/ovidweb.cgi?&S=CGHBPDIOHLHFAOPAFNIKOEEGGLDHAA00&SELECT=S.sh%7c&R=2&Process+Action=display)  [More](http://ovidsp.uk.ovid.com/sp-3.19.0a/ovidweb.cgi?&S=CGHBPDIOHLHFAOPAFNIKOEEGGLDHAA00&SELECT=S.sh%7c&Expand=1&Main+Search+Page=Main+Search+Page) |  |
|  | 3 | 1 or 2 | 10227 | Advanced | [Display Results](http://ovidsp.uk.ovid.com/sp-3.19.0a/ovidweb.cgi?&S=CGHBPDIOHLHFAOPAFNIKOEEGGLDHAA00&SELECT=S.sh%7c&R=3&Process+Action=display)  [More](http://ovidsp.uk.ovid.com/sp-3.19.0a/ovidweb.cgi?&S=CGHBPDIOHLHFAOPAFNIKOEEGGLDHAA00&SELECT=S.sh%7c&Expand=1&Main+Search+Page=Main+Search+Page) |  |
|  | 4 | ethnic*.mp. | 6662 | Advanced | [Display Results](http://ovidsp.uk.ovid.com/sp-3.19.0a/ovidweb.cgi?&S=CGHBPDIOHLHFAOPAFNIKOEEGGLDHAA00&SELECT=S.sh%7c&R=4&Process+Action=display)  [More](http://ovidsp.uk.ovid.com/sp-3.19.0a/ovidweb.cgi?&S=CGHBPDIOHLHFAOPAFNIKOEEGGLDHAA00&SELECT=S.sh%7c&Expand=1&Main+Search+Page=Main+Search+Page) |  |
|  | 5 | Asian.mp. or Asian Continental Ancestry Group/ | 5562 | Advanced | [Display Results](http://ovidsp.uk.ovid.com/sp-3.19.0a/ovidweb.cgi?&S=CGHBPDIOHLHFAOPAFNIKOEEGGLDHAA00&SELECT=S.sh%7c&R=5&Process+Action=display)  [More](http://ovidsp.uk.ovid.com/sp-3.19.0a/ovidweb.cgi?&S=CGHBPDIOHLHFAOPAFNIKOEEGGLDHAA00&SELECT=S.sh%7c&Expand=1&Main+Search+Page=Main+Search+Page) |  |
|  | 6 | black.mp. or African Continental Ancestry Group/ | 4821 | Advanced | [Display Results](http://ovidsp.uk.ovid.com/sp-3.19.0a/ovidweb.cgi?&S=CGHBPDIOHLHFAOPAFNIKOEEGGLDHAA00&SELECT=S.sh%7c&R=6&Process+Action=display)  [More](http://ovidsp.uk.ovid.com/sp-3.19.0a/ovidweb.cgi?&S=CGHBPDIOHLHFAOPAFNIKOEEGGLDHAA00&SELECT=S.sh%7c&Expand=1&Main+Search+Page=Main+Search+Page) |  |
|  | 7 | Ethnic Groups/ or multiethnic.mp. | 858 | Advanced | [Display Results](http://ovidsp.uk.ovid.com/sp-3.19.0a/ovidweb.cgi?&S=CGHBPDIOHLHFAOPAFNIKOEEGGLDHAA00&SELECT=S.sh%7c&R=7&Process+Action=display)  [More](http://ovidsp.uk.ovid.com/sp-3.19.0a/ovidweb.cgi?&S=CGHBPDIOHLHFAOPAFNIKOEEGGLDHAA00&SELECT=S.sh%7c&Expand=1&Main+Search+Page=Main+Search+Page) |  |
|  | 8 | 4 or 5 or 6 or 7 | 15398 | Advanced | [Display Results](http://ovidsp.uk.ovid.com/sp-3.19.0a/ovidweb.cgi?&S=CGHBPDIOHLHFAOPAFNIKOEEGGLDHAA00&SELECT=S.sh%7c&R=8&Process+Action=display)  [More](http://ovidsp.uk.ovid.com/sp-3.19.0a/ovidweb.cgi?&S=CGHBPDIOHLHFAOPAFNIKOEEGGLDHAA00&SELECT=S.sh%7c&Expand=1&Main+Search+Page=Main+Search+Page) |  |
|  | 9 | 3 and 8 | 261 | Advanced | [Display Results](http://ovidsp.uk.ovid.com/sp-3.19.0a/ovidweb.cgi?&S=CGHBPDIOHLHFAOPAFNIKOEEGGLDHAA00&SELECT=S.sh%7c&R=9&Process+Action=display)  [More](http://ovidsp.uk.ovid.com/sp-3.19.0a/ovidweb.cgi?&S=CGHBPDIOHLHFAOPAFNIKOEEGGLDHAA00&SELECT=S.sh%7c&Expand=1&Main+Search+Page=Main+Search+Page) |  |
|  | 10 | Grounded Theory/ | 1 | Advanced | [Display Results](http://ovidsp.uk.ovid.com/sp-3.19.0a/ovidweb.cgi?&S=CGHBPDIOHLHFAOPAFNIKOEEGGLDHAA00&SELECT=S.sh%7c&R=10&Process+Action=display)  [More](http://ovidsp.uk.ovid.com/sp-3.19.0a/ovidweb.cgi?&S=CGHBPDIOHLHFAOPAFNIKOEEGGLDHAA00&SELECT=S.sh%7c&Expand=1&Main+Search+Page=Main+Search+Page) |  |
|  | 11 | Interview/ or interview*.mp. | 17176 | Advanced | [Display Results](http://ovidsp.uk.ovid.com/sp-3.19.0a/ovidweb.cgi?&S=CGHBPDIOHLHFAOPAFNIKOEEGGLDHAA00&SELECT=S.sh%7c&R=11&Process+Action=display)  [More](http://ovidsp.uk.ovid.com/sp-3.19.0a/ovidweb.cgi?&S=CGHBPDIOHLHFAOPAFNIKOEEGGLDHAA00&SELECT=S.sh%7c&Expand=1&Main+Search+Page=Main+Search+Page) |  |
|  | 12 | content analysis.mp. | 413 | Advanced | [Display Results](http://ovidsp.uk.ovid.com/sp-3.19.0a/ovidweb.cgi?&S=CGHBPDIOHLHFAOPAFNIKOEEGGLDHAA00&SELECT=S.sh%7c&R=12&Process+Action=display)  [More](http://ovidsp.uk.ovid.com/sp-3.19.0a/ovidweb.cgi?&S=CGHBPDIOHLHFAOPAFNIKOEEGGLDHAA00&SELECT=S.sh%7c&Expand=1&Main+Search+Page=Main+Search+Page) |  |
|  | 13 | Focus Groups/ or focus group*.mp. | 1288 | Advanced | [Display Results](http://ovidsp.uk.ovid.com/sp-3.19.0a/ovidweb.cgi?&S=CGHBPDIOHLHFAOPAFNIKOEEGGLDHAA00&SELECT=S.sh%7c&R=13&Process+Action=display)  [More](http://ovidsp.uk.ovid.com/sp-3.19.0a/ovidweb.cgi?&S=CGHBPDIOHLHFAOPAFNIKOEEGGLDHAA00&SELECT=S.sh%7c&Expand=1&Main+Search+Page=Main+Search+Page) |  |
|  | 14 | discourse*.mp. | 165 | Advanced | [Display Results](http://ovidsp.uk.ovid.com/sp-3.19.0a/ovidweb.cgi?&S=CGHBPDIOHLHFAOPAFNIKOEEGGLDHAA00&SELECT=S.sh%7c&R=14&Process+Action=display)  [More](http://ovidsp.uk.ovid.com/sp-3.19.0a/ovidweb.cgi?&S=CGHBPDIOHLHFAOPAFNIKOEEGGLDHAA00&SELECT=S.sh%7c&Expand=1&Main+Search+Page=Main+Search+Page) |  |
|  | 15 | ethnograph*.mp. | 144 | Advanced | [Display Results](http://ovidsp.uk.ovid.com/sp-3.19.0a/ovidweb.cgi?&S=CGHBPDIOHLHFAOPAFNIKOEEGGLDHAA00&SELECT=S.sh%7c&R=15&Process+Action=display)  [More](http://ovidsp.uk.ovid.com/sp-3.19.0a/ovidweb.cgi?&S=CGHBPDIOHLHFAOPAFNIKOEEGGLDHAA00&SELECT=S.sh%7c&Expand=1&Main+Search+Page=Main+Search+Page) |  |
|  | 16 | ethnonursing.mp. | 1 | Advanced | [Display Results](http://ovidsp.uk.ovid.com/sp-3.19.0a/ovidweb.cgi?&S=CGHBPDIOHLHFAOPAFNIKOEEGGLDHAA00&SELECT=S.sh%7c&R=16&Process+Action=display)  [More](http://ovidsp.uk.ovid.com/sp-3.19.0a/ovidweb.cgi?&S=CGHBPDIOHLHFAOPAFNIKOEEGGLDHAA00&SELECT=S.sh%7c&Expand=1&Main+Search+Page=Main+Search+Page) |  |
|  | 17 | phenomenological*.mp. | 111 | Advanced | [Display Results](http://ovidsp.uk.ovid.com/sp-3.19.0a/ovidweb.cgi?&S=CGHBPDIOHLHFAOPAFNIKOEEGGLDHAA00&SELECT=S.sh%7c&R=17&Process+Action=display)  [More](http://ovidsp.uk.ovid.com/sp-3.19.0a/ovidweb.cgi?&S=CGHBPDIOHLHFAOPAFNIKOEEGGLDHAA00&SELECT=S.sh%7c&Expand=1&Main+Search+Page=Main+Search+Page) |  |
|  | 18 | qualitative*.mp. or Qualitative Research/ | 8449 | Advanced | [Display Results](http://ovidsp.uk.ovid.com/sp-3.19.0a/ovidweb.cgi?&S=CGHBPDIOHLHFAOPAFNIKOEEGGLDHAA00&SELECT=S.sh%7c&R=18&Process+Action=display)  [More](http://ovidsp.uk.ovid.com/sp-3.19.0a/ovidweb.cgi?&S=CGHBPDIOHLHFAOPAFNIKOEEGGLDHAA00&SELECT=S.sh%7c&Expand=1&Main+Search+Page=Main+Search+Page) |  |
|  | 19 | 10 or 11 or 12 or 13 or 14 or 15 or 16 or 17 or 18 | 24544 | Advanced | [Display Results](http://ovidsp.uk.ovid.com/sp-3.19.0a/ovidweb.cgi?&S=CGHBPDIOHLHFAOPAFNIKOEEGGLDHAA00&SELECT=S.sh%7c&R=19&Process+Action=display)  [More](http://ovidsp.uk.ovid.com/sp-3.19.0a/ovidweb.cgi?&S=CGHBPDIOHLHFAOPAFNIKOEEGGLDHAA00&SELECT=S.sh%7c&Expand=1&Main+Search+Page=Main+Search+Page) |  |
|  | 20 | 9 and 19 | 86 | Advanced |  |  |

**TOTAL = 999**

**DUPLICATES = 249**

**To be screened (title/abstract) = 750**
