# Supplementary material for: Barriers and facilitators in accessing dementia care by ethnic minority groups: a meta-synthesis of qualitative studies
Source: BMC Psychiatry. 2017 Aug 30;17:316. doi: 10.1186/s12888-017-1474-0 (PMC5577676; doi:10.1186/s12888-017-1474-0)
Supplement: Supplementary file 2 — Table showing the original themes around barriers extracted from papers with recoding to new concepts. Table showing the themes extracted from papers along with how they were ordered and recoded and how they were then grouped under new overarching concepts. (DOCX 20 kb) [file 12888_2017_1474_MOESM2_ESM.docx]

Additional file 2

Table showing the original themes around barriers extracted from papers with recoding to new concepts

| **BARRIERS** |  |  |
| --- | --- | --- |
| **Original themes (paper numbers)** | **New concept** | **Overarching  concept** |
| Lack of knowledge – disease (4,6,11,16,28) Lack of understanding- causes/symptoms (4,5,6,11,16,19,22,26,30) Normalisation of dementia as old age (5,6,9,11,14,19,23,26,30) Ageing= time of isolation & withdrawal, cognitive decline hidden (16) | Lack of carer/patient understanding of the causes and symptoms of dementia |  |
| Lack of understanding of services eg respite (5) Lack of knowledge/familiarity with services (16,18,20,22,24,29) Lack of info on legalities and function of carer role (17) Difficult to find out how care system works and how to find services (24) Difficulty manoeuvring complex bureaucracies (5,11) Community/welfare services don’t exist or very different in country of origin (24) Language barriers to accessing/receiving care (3,9,17,24,28) Language barriers to info/awareness of services (5,17) | Lack of Knowledge, familiarity and awareness of services and how to navigate the system |  |
| Lack of specialised dementia knowledge in practitioners (11,24) Failure to diagnose (GP) (5,23) Attitudes of health professionals & inability to pick up cognitive impairment (22) Not providing a diagnosis in a way that can be understood (4) GPs lack linguistic & cultural ability to achieve diagnosis (10) Historical pervasive racism leading to misdiagnosis (10) | Professionals lack of specialist knowledge and lack of cultural and linguistic ability to achieve diagnosis | **Inadequacies** |
| Small number of potential service users & geographical dispersal = access issues (10) Shortage of services/waiting lists (10,24,26) Lack of choice/limitations in services (10,11) Structure of services=unhelpful/unusable (2) Lack of understanding of diversity within cultures (26) Low levels of literacy= isolation (5,29) Lack of understanding of cultural needs (3) Lack of cultural awareness/needs (3,9) Marginalisation of ethno-specific services within system (10) | Limitations of available services and lack of cultural  awareness |  |
| View that little can be done to treat dementia (6,14) Perception practitioners see it as untreatable (10) Not seen as needing medical care (5,9,13,14,24) Not clear why going to Dr would be helpful (19) | Preconceived ideas of treatability |  |
| Anxiety about others providing care (5) Mistrust & social mores about accepting white carers (5) Difficulties having outsiders come in to home (24) Moral obligation to family/familial responsibility (15,22,14,30) Belief that family care is better (5) Gender issues- personal care by opposite sex, sitting in room or sleeping in same room= incompatible with faith (24) Personal care even by same sex can be unacceptable (24) | Anxiety/trust issues about ‘outside’ support and its cultural  appropriateness |  |
| Mistrust of services/covert racism (13,22) Institutionalised racism (28,29) Perception of negative care provider attitudes (7) Concerns over immigration status (11) Fear of retaliation if speak up to authorities (11) | Issues around trust and racism, both historic and  current |  |
| Pervasive stigma around mental illness/dementia (6,14,16,22,26,30) Don’t want diagnosis due to stigma (9,28) Fear of upsetting/betraying pt by seeking help (22) Hiding diagnosis from community/reluctance to seek help (17,20,25,27) Confidentiality (fear ppl will find out) (9) Coping strategies designed to conceal dementia (15) Denial by pt they have problem/refusal to accept help (22) Patients are isolated from family and community life (6) | Societal stigma of mental illness and denial or concealment as a defence against the reactions of others |  |
| Folk beliefs- (5,11,15,30) Extreme respect for authority prevents asking questions (14) Perceived conflict between Asian & western medicine (5,14) Preference for folk/home remedies (5) Residential care= ideologically unacceptable & culturally inappropriate (6) Culturally appropriate food (24) Perception ppl should overcome own problems (6) Struggling to reconcile multiple values & commitments (20) Assumption that Asian families ‘look after their own’ (9) | Cultural issues impacting on perceptions of Western medicine and the acceptability of services | **Cultural habitus/ experiences** |
| Help seeking= failure to fulfil role/responsibilities (18) Guilt about using services (5) View of support services as welfare/handout (=poverty) (5,24,29) Seeking help seen as complaining about familial duty (5) Nursing home= breach of familial duty (11,27) Balance between shame & inner pride (15) Reactions of others in community informs decisions about care (20) Guilt/pressure to provide care at home (24) Expectation to cope in stress & adversity (29) Decision making/gender issues/family conflict (2,11,26) | The impact of cultural/familial expectations and community perceptions on care decisions |  |
| Dementia only picked up if family raise it/persistence needed (13) Health care professionals not listening/ dismissive/ trivialising carer concerns (22,23,26,30) Excluding carers from appointments/correspondence (22) Likened to battle in which constantly fighting for info/advice/support (18) Disillusionment with GPs & exclusion from services (16) Impression GP could not/would not help (16) | Health care provider exclusion and dismissal of carer concerns/Negative carer experiences of help seeking |  |
| Guilt (5)  Denial (5,14,17)  Fear (5,20,21)  Embarrassment (5,9,20,25)  Shame (14,20) | Negative emotions associated with response from own community |  |

Table showing the original themes around facilitators extracted from papers with recoding to new concepts

| **FACILITATORS** |  |  |
| --- | --- | --- |
| **Original themes (paper numbers)** | **New concept** | **Overarching  concept** |
| Educated carers=less stressed (20) educate community groups to pass on knowledge (26) early education – after onset (26) education & information needed (26) Providing info in native language (11,24) speaking language not enough (25) GPs that take the time to educate (19) Staff to refer, support and educate (7) | Improving knowledge of dementia |  |
| Firm belief in dr-pt relationship (1) Once participating in main stream services removes some mistrust (5) Trust & confidence building (5) prepared to use services when under stress and comfortable with the service (24) | Tackling the issue of 'outsiders' | **Education &  addressing community perceptions** |
| Pervasive stigma around mental illness/dementia (6,14,16,22,26,30) Don’t want diagnosis due to stigma (9,28) Fear of upsetting/betraying pt by seeking help (22) emphasis on physical rather than mental (30) Community acceptance of service (5) Importance of social networks in getting diagnosis (7) networking and peer support (25, 28) Reduce stigma -naming services clubs (5) normalising help seeking (30) Address stigma of accepting help (6) Present as physical rather than mental- remove stigma (6) Medicalise staff= more official/respect for med practitioners (6) Dispel myths/folk beliefs (5) Educate whole community (5) | Addressing societal stigma/denial/ concealment |  |
| continuity of care (22) time to build a relationship (24) slow introduction to services (24) building trust (25) | Continuity of care |  |
| staff training-culture (24,26) education of service providers-disease (26) services that consider culture=more successful (3) Importance of how services are introduced/offered (5) Education of professionals about dementias and also to be culturally sensitive (6) basic communication training for non-language speakers (24) | Improving training for practitioners around  identifying and screening for dementia | **Training & addressing  services issues** |
| Cultural adaptations-food/activities (5, 24) Cultural adaptation- combine groups with meals (5) Cultural adapt- respect (5) Cultural adapt- family values (5) Carers from same community (5) Cultural adapt- avoid stereotyping (6) Augmenting home care- Balance between family and support (15) preference for culturally adapted services (24) cultural understanding (25) more flexible provision to fit culture (25) Some cultures v respectful of GPs = use this (14) bilingual GPs and specialists (23) same language background workers (24) Would use care if prevented residential care (11) Non-institutional settings (5) | Broadening and adapting services |  |
